# The E6 Oncoprotein of HPV16 AA-c Variant Regulates Cell Migration through the MINCR/miR-28-5p/RAP1B Axis

**DOI:** 10.3390/v14050963

**Published:** 2022-05-05

**Authors:** Eduardo Gil Perez-Bacho, Fredy Omar Beltrán-Anaya, Elena Arechaga-Ocampo, Daniel Hernández-Sotelo, Olga Lilia Garibay-Cerdenares, Berenice Illades-Aguiar, Luz Del Carmen Alarcón-Romero, Oscar Del Moral-Hernández

**Affiliations:** 1 Laboratorio de Virología, Facultad de Ciencias Químico Biológicas, Universidad Autónoma de Guerrero, Chilpancingo 39070, Mexico; edbach1191@gmail.com (E.G.P.-B.); frebeltran@hotmail.com (F.O.B.-A.); 2 Departamento de Ciencias Naturales, Universidad Autónoma Metropolitana Unidad Cuajimalpa, Mexico City 05300, Mexico; earechaga@cua.uam.mx; 3 Laboratorio de Epigenética del Cáncer, Facultad de Ciencias Químico Biológicas, Universidad Autónoma de Guerrero, Chilpancingo 39070, Mexico; dhernandez@uagro.mx; 4 Laboratorio de Biomedicina Molecular, Facultad de Ciencias Químico Biológicas, Universidad Autónoma de Guerrero, Chilpancingo 39070, Mexico; olgaribayce@conacyt.mx (O.L.G.-C.); billades@uagro.mx (B.I.-A.); 5 CONACyT-Universidad Autónoma de Guerrero, Chilpancingo 39070, Mexico; 6 Laboratorio de Citopatología e Histoquímica, Facultad de Ciencias Químico Biológicas, Universidad Autónoma de Guerrero, Chilpancingo 39070, Mexico; lcalarcon@uagro.mx

**Keywords:** lncRNAs, MINCR, miR-28-5p, RAP1B, E6 oncoprotein, HPV16 AA-c variant

## Abstract

The E6 oncoprotein of HPV16 variants differentially alters the transcription of the genes involved in migration and non-coding RNAs such as lncRNAs. The role of the lncRNA MINCR in cervical cancer and its relationship with variants of oncogenic HPV remain unknown. Therefore, the objective of this study was to analyze the effect of the E6 oncoprotein of the AA-c variant of HPV16 in cell migration through the MINCR/miR-28-5p/RAP1B axis. To explore the functional role of MINCR in CC, we used an in vitro model of C33-A cells with exogenous expression of the E6 oncoprotein of the AA-c variant of HPV16. Interfering RNAs performed MINCR silencing, and the expression of miR-28-5p and RAP1B mRNA was analyzed by RT-qPCR. We found that C33-A/AA-c cells expressed MINCR 8-fold higher compared to the control cells. There is an inverse correlation between the expression of miR-28-5p and RAP1B in C33-A/AA-c cells. Our results suggest that MINCR might regulate the expression of RAP1B through the inhibition of miR-28-5p in CC cells expressing the E6 oncoprotein of HPV16 AA-c. We report, for the first time, that the MINCR/miR-28-5p/RAP1B axis positively regulates cell migration in CC-derived cells that express the E6 oncoprotein of the AA-c variant of HPV16.

## 1. Introduction

Cervical cancer (CC) is one of the most common gynecological tumors and is the fourth leading cause of cancer-related deaths in women worldwide [1] (https://gco.iarc.fr/, accessed on 9 December 2021). There are different risk factors for the development of CC, but the main cause is persistent infection with high-risk human papillomavirus (HR-HPV) [2]. More than 200 HPV genotypes have been described, which are classified according to their oncogenic risk for the development of CC. Among the genotypes of HR-HPV, type 16 (HPV16) is the most frequent worldwide [3].

The E6 and E7 oncoproteins of HR-HPV are responsible for inducing carcinogenesis, and their continuous expression is necessary for the growth of tumor cells [4,5]. Analysis of the E6 gene sequence has shown the existence of intratypical variants of HPV16, which are classified into five major variant lineages: European (EUR), Asian (As), Asian–American (AA), and two African lineages, African-1 and African-2 (AFR1 and AFR2) [6]. AA variants have been found more frequently in CC tissue, and functional assays have demonstrated that the E6 oncoprotein of the AA-c variant alone is sufficient to immortalize cells and increase cell migration [7]. Therefore, it is necessary to explore the molecular mechanisms by which HPV16 variants modulate their oncogenic potential in the development of CC.

The HPV16 E6 oncoprotein can bind to a wide variety of cellular proteins and thus interfere in cellular processes important for the development of cancer. Through the binding of E6AP ubiquitin ligase, the E6 oncoprotein induces degradation of the tumor suppressor p53, and therefore HPV16-positive cells show chromosomal instability [8]. In addition to direct interaction with cellular proteins, E6 is capable of indirectly altering its expression through different mechanisms. It has previously been reported that the expression of the E6 oncoprotein of HPV16 variants differentially alters the transcription of the genes involved in cell proliferation, adhesion, and migration [9]. This effect on the cellular transcriptome is not limited to coding genes, since genes that code for non-coding RNAs (ncRNAs) such as long non-coding RNAs (lncRNAs) are also affected. LncRNAs are molecules involved in epigenetic regulation and other biological processes; therefore, by altering their expression levels, they favor carcinogenesis in several types of tumors, including CC [10,11]. The expression of some lncRNAs in CC and their effect on carcinogenesis have been evaluated; for example, it has been observed that the overexpression of HOTAIR increases cell proliferation, migration, and invasion through the activation of the Notch signaling pathway [12]. Additionally, the overexpression of lnc-FANCI-2 in cervical cancer CaSki cells has been observed, where lnc-FANCI-2 is induced primarily by the E7 protein by interacting with the cellular transcription factor YY1 and, to a lesser extent, by E6 [13]. In addition to lncRNAs, HPV also alters the expression of other ncRNAs such as miRNAs, and miR-18a, which directly targets mRNA of the Hippo pathway regulatory kinase STK4, has been found overexpressed in cervical cancer cell lines in which STK4 is downregulated [14].

The function of lncRNAs during cellular transformation is not yet fully understood; however, it has been reported that they can act as molecular scaffolds, guides for transcription factors, decoys for other molecules, stabilizers of mRNAs, and endogenous competitor RNAs (ceRNAs)—functions that have been extensively revised [15]. ceRNAs act as molecular sponges of microRNAs (miRNAs) and consequently inhibit the regulatory action on their target mRNAs [16].

MINCR is an lncRNA that is overexpressed in some types of tumors, where its silencing has been found to suppress cell proliferation and migration [17]. MINCR has been reported to upregulate proteins such as β-catenin through the inhibition of miRNAs such as miR-708-5p, which results in the activation of the WNT/β-catenin signaling pathway [18].

Ras-associated protein 1 (RAP1) is a protein of the Ras family of small G proteins, which regulates some pathways involved in proliferation, differentiation, polarity, and apoptosis. This protein has two highly homologous isoforms, RAP1A and RAP1B, which are products of two different genes located on chromosomes 1 and 12, respectively [19]. The role of RAP1B in cancer is not entirely clear, but some miRNAs that target RAP1B have been found to inhibit cell migration, invasion, and metastasis in various types of tumors, such as miR-100 in colorectal cancer or miR-149 in lung adenocarcinoma [20,21]. The importance of ceRNAs in cancer progression has been demonstrated. However, the role of MINCR in cervical cancer and its relationship with the variants of oncogenic HPV remain unknown; therefore, the objective of this study was to analyze the effect of the E6 oncoprotein of the AA-c variant of HPV16 on the expression of RAP1B through the lncRNA MINCR.

## 2. Materials and Methods

### 2.1. Screening of Differentially Expressed lncRNAs

The raw expression data of the C33-A cells containing the E6 variant of HPV16 were downloaded from the Gene Expression Omnibus repository (GEO, GSE73761). All data (CEL files) were analyzed with Transcriptome Analysis Console (TAC, v.4.0) software as follows: (i) fluorescence intensities were normalized by the Robust Multi-chip Analysis-SST algorithm; (ii) differentially expressed genes (DEGs) were defined by a rate of change > 1.5 and a *p*-value < 0.05; (iii) DEGs derived between the mock and C33-A experimental conditions were subtracted from the DEGs in comparisons of the mock and C33-A/E6 variants; (iv) probe IDs from the microarray were used to identify differentially expressed lncRNAs. Finally, using the fluorescence values, heat maps were constructed in the R environment.

### 2.2. Bioinformatic Analysis

MINCR target miRNAs were predicted through the starBase platform (http://starbase.sysu.edu.cn/index.php, accessed on 15 June 2020). Subsequently, data extracted from TCGA (https://portal.gdc.cancer.gov/projects/TCGA-CESC, accessed on 17 June 2020) were used to evaluate the expression of these miRNAs in CC and to correlate their expression with MINCR expression. Using the TargetScan (http://www.targetscan.org/vert_71/, accessed on 22 June 2020), miRDB (http://mirdb.org/, accessed on 22 June 2020), and miRTarBase (https://mirtarbase.cuhk.edu.cn/, accessed on 22 June 2020) platforms, the targets of the miRNAs whose expression was negatively correlated with MINCR expression were searched.

### 2.3. Cell Culture and Transfection of siRNAs

C33-A cells transfected with the E6 gene of the AA-c variant of HPV16 [9] were cultured in DMEM medium (Invitrogen, Carlsbad, CA, USA) supplemented with 10% FBS, 1% penicillin/streptomycin, and 1% geneticin (Gibco, San Jose, CA, USA) and then incubated at a temperature of 37 °C and 5% CO_2_. For the silencing of MINCR, 1 × 10^6^ cells/well were seeded in a 6-well plate, where the cells were transfected with 5 pmol of Silencer Select siRNA or Silencer Select Negative Control siRNA (Invitrogen, Carlsbad, CA, USA) using Lipofectamine 3000 (Invitrogen, Carlsbad, CA, USA). The transfectant was removed after 4 h, and the cells were incubated in serum-free DMEN medium for 24 h post-transfection.

### 2.4. RNA Extraction

Total RNA was obtained from cell cultures using TRIzol (Invitrogen, Carlsbad, CA, USA) according to the manufacturer’s instructions. The RNA was resuspended in nuclease-free water to evaluate its purity and concentration in a NanoDrop 2000 Kit (Thermo Scientific, Carlsbad, CA, USA). Then, the RNA samples were treated with DNase I (Invitrogen, Carlsband, CA, USA).

### 2.5. RT-qPCR

cDNA was obtained from total RNA using the enzyme SuperScript III and oligo dT (Invitrogen, Carlsbad, CA, USA) for the analysis of RAP1B mRNA expression. qPCR was carried out with Sybr Green (Applied Biosystems, Carlsbad, CA, USA) using the primers F: 5’ TCC ATC ACA gCA CAg TCC AC 3’ and R: 5’ AAT TTG CCG CAC TAG GTC AT 3’ for RAP1B mRNA, and primers F: 5’ GAC CCC TTC ATT GAC CTC AAC 3’ and R: 5’ GTG GCA GTG ATG GCA TGG AC 3’ for GAPDH mRNA as an endogenous control. For analysis of the expression of miR-28-5p, reverse transcription was performed using a TaqMan MicroRNA Reverse Transcription Kit (Applied Biosystems, Carlsbad, CA, USA) and TaqMan probes (Applied Biosystems, Carlsbad, CA, USA). RNU44 snoRNA was used as an endogenous control. Relative expression values were obtained using the 2^−ΔΔCt^ method. Three independent experiments were performed in triplicate.

### 2.6. Western Blot

Total proteins were extracted from cell cultures using RIPA extraction buffer (Thermo Scientific, Carlsbad, CA, USA) supplemented with protease and phosphatase inhibitors (Thermo Scientific, Carlsbad, CA, USA). The proteins were separated through SDS-PAGE (12.5%) and subsequently transferred to nitrocellulose membranes, which were blocked with 5% skimmed milk and incubated with the primary anti-RAP1B antibody (CST, Danvers, MA, USA) at a 1:1000 dilution overnight at 4 °C. After washing with TBS-T, the membranes were incubated with anti-Rabbit secondary antibody coupled to HRP (Invitrogen, Carlsbad, CA, USA) at a 1:2000 dilution. An antibody against GAPDH was used as an endogenous control. The membranes were reused and incubated with primary anti-GAPDH antibody at a 1:2000 dilution (Invitrogen, Carlsbad, CA, USA) overnight at 4 °C. Pierce ECL Western Blotting Substrate (Thermo Scientific, Carlsbad, CA, USA) was used for development.

### 2.7. Cell Migration Assays

The migration assay was carried out by seeding 2.5 × 10^5^ cells in the upper chamber layer of a transwell system with serum-free DMEM medium, while supplemented DMEM medium with 10% FBS was placed in the bottom chamber. The chambers were incubated at 37 °C with 5% CO_2_ for 48 h. The cells were fixed with 4% paraformaldehyde and stained with crystal violet. Cells were quantified using ImageJ software 4 (https://imagej.nih.gov/ij/, accessed on 17 May 2021).

### 2.8. Statistical Analysis

The data were analyzed using STATA version 14 software (StataCorp LP, 2015). Student’s *t*-test was applied to normally distributed data, while the Mann–Whitney–Wilcoxon test was applied to the rest. A *p*-value of ≤0.05 was considered significant.

## 3. Results

### 3.1. The E6 Oncoprotein of the HPV16 AA-c Variant Increases the Expression Levels of lncRNA MINCR in Cells Derived from Cervical Cancer

**Figure 1:**
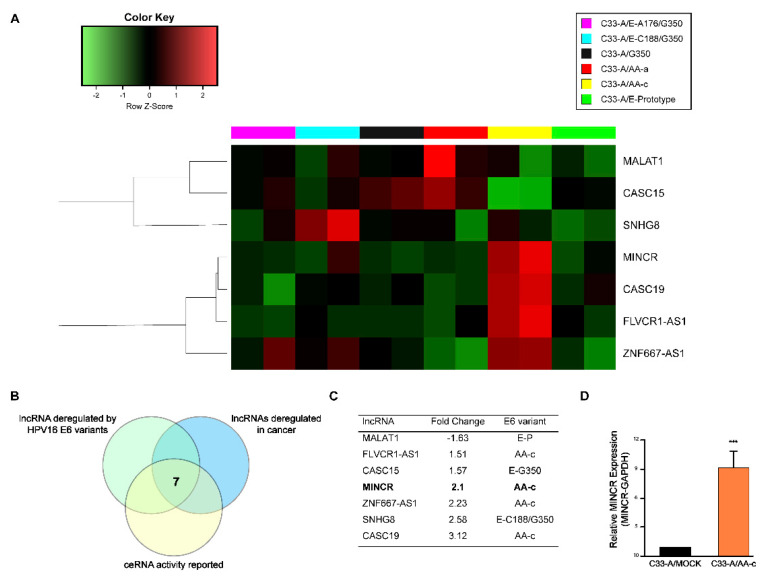
The AA-c variant alters the expression of MINCR and other lncRNAs with activity as a ceRNA. (**A**) Heat map illustrating that lncRNAs are differentially expressed in C33-A cells expressing HPV16 variants. (**B**,**C**) lncRNAs filtered after applying the selection criteria; (**D**) MINCR expression in C33-A/AA-c cells determined by RT-qPCR. Relative expression was normalized to the GAPDH gene. Student’s *t*-test, *** *p* < 0.001.

In order to observe the effect of the E6 oncoprotein of the HPV16 variants on the expression profile of lncRNAs in CC cells, microarray assays were carried out in C33-A cells transfected with the variants of the oncogene E6 [9] (Figure 1A). The results show that among the lncRNAs differentially expressed, seven lncRNAs had been previously reported to have the activity of ceRNAs and to be altered in cancer (Figure 1B). Of them, MINCR had not previously been reported in CC; moreover, MINCR was overexpressed in cells transfected with the oncogene E6 of the AA-c variant (C33-A/AA-c) (Figure 1C). To validate the results of the microarray, the expression of the LncRNA MINCR was determined by real-time RT-PCR in C33-A cells that express the E6 oncoprotein of the E-prototype, and variants AA-a, AA-c, E-G350, and E-A176/G350 (data not shown), and it was found that, in C33-A/AA-c cells, the expression of MINCR was 8-fold higher compared to that in the control cells (Figure 1D). The AA-c variant was chosen because it expresses the highest levels of MINCR and because Asian–American HPV16 variants have previously been reported to have greater oncogenic potential compared to European variants [22].

### 3.2. lncRNA MINCR Expression Levels Correlate with miR-28-5p Levels

**Figure 2:**
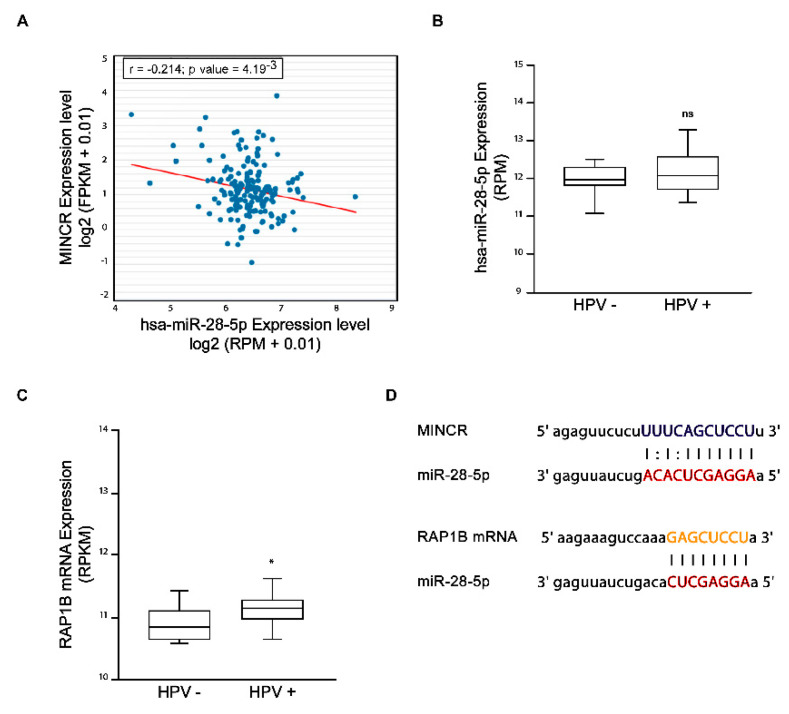
Bioinformatic analysis for the construction of the MINCR/miR-28-5p/RAP1B interaction axis built from data obtained from the TCGA database. (**A**) Correlation between MINCR and miR-28-5p expression levels. (**B**) Expression of miR-28-5p in patients with CC. (**C**) Expression of RAP1B mRNA in patients with DC. (**D**) Base pairing prediction for miR-28-p with MINCR and with RAP1B mRNA. The sequences were obtained using the TargetScanHuman platform; Mann–Whitney–Wilcoxon test, * *p* ≤ 0.05.

To identify the possible axis of interaction of MINCR with miRNA and its target messenger, a bioinformatic analysis was performed using the StarBase database for the construction of the interaction axis of lncRNAs–miRNAs–mRNA. The results show 12 miRNAs with a potential role in the interaction with MINCR (Table S1). miRNA sets were analyzed in several tumor samples obtained from the TCGA database to correlate their expression levels with the levels of MINCR. A negative correlation between the expression of MINCR and miR-28-5p in patients with pancreatic adenocarcinoma was found, suggesting the role of MINCR as a ceRNA for miR-28-5p (Figure 2A). Subsequently, prediction of the target genes of miR-28-5p was performed using the TargetScan, miRDB, and miRTarBase databases. The results show three genes coincident in the three platforms (Table S2); among them, the RAP1B gene has a role in carcinogenesis due to its participation in cellular processes such as cell migration and invasion. Direct interaction between RAP1B mRNA and miR-28-5p has already been demonstrated in endometrial cancer cells [16]. In order to evaluate whether the MINCR/miR-28-5p/RAP1B interaction axis might be altered during HPV16-induced carcinogenesis (Figure 2B,C), TCGA data were used to verify the miR-28-5p and RAP1B expression levels in patients CC-positive for HPV16 and were compared to patients negative for the virus. RAP1B levels were significantly higher in those patients with HPV16 infection, but no significant differences were found in the expression levels of miR-28-5p. These results suggest that other mechanisms might regulate the abundance of the RAP1B protein in addition to miR-28-5p. Additionally, it should be considered that the data used came from clinical samples with a highly heterogeneous composition, including the presence of HPV16 variants. Subsequently, the TargetScanHuman 7.1 platform was used to search for the miR-28-5p binding sites in MINCR and RAP1B mRNA. Interestingly, a similar sequence was found in both transcripts (Figure 2D), which could suggest a competition between MINCR and RAP1B mRNA for binding to miR-28-5p.

### 3.3. The Expression of miR-28-5p and RAP1B mRNA Is Altered in C33-A/AA-c Cells

**Figure 3:**
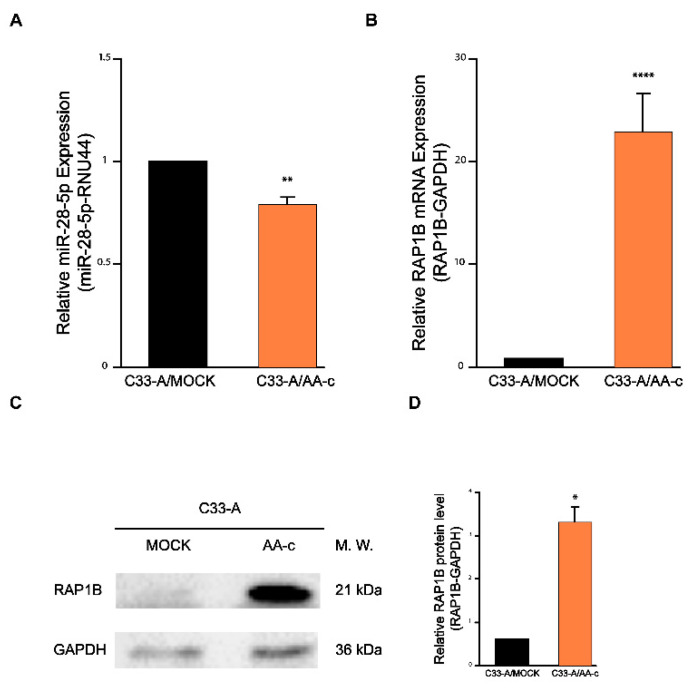
Expression of miR-28-5p and RAP1B in C33-A/AA-c cells. (**A**,**B**) RT-qPCR was used to measure the level of miR-28-5p and RAP1B mRNA in C33-A/AA-c cells compared to control cells. The relative expression was normalized with the small nucleolar RNA RNU44 and GAPDH, respectively. (**C**) RAP1B protein level measured by Western blot. The relative levels of RAP1B obtained with respect to GAPDH protein levels were used as an endogenous control. (**D**) Densitometry analysis is shown. Student’s *t*-test * *p* ≤ 0.05, ** *p* < 0.01, **** *p* < 0.0001.

To explore the role of the variant AA-c in the MINCR/miR-28-5p/RAP1B axis, the expression of miR-28-5p and RAP1B was evaluated in C33-A/AA-c cells compared to the control C33-A/MOCK cells to correlate with the results previously observed for MINCR from the microarrays. The results show that the expression levels of miR-28-5p were 0.3-fold lower in C33-A/AA-c cells compared to the control (Figure 3A). In contrast, the expression levels of RAP1B mRNA and protein expression were 21.8- and 2.3-fold higher in C33-A/AA-c cells, respectively (Figure 3B–D). These results suggest that there is an inverse correlation between the expression of miR-28-5p and RAP1B in C33-A/AA-c cells.

### 3.4. The MINCR/miR-28-5p/RAP1B Axis Regulates Cell Migration in C33-A/AA-c Cells

**Figure 4:**
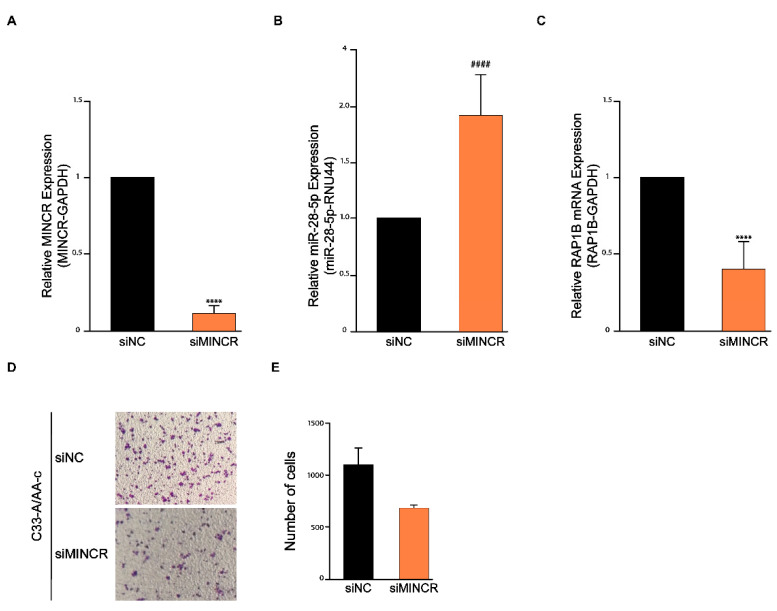
MINCR regulates migration in C33-A/AA-c cells through modulating the expression of miR-28-5p and RAP1B. (**A**) Expression of MINCR after 24 h post-transfection of C33-A/AA-c cells with 5 pmol of siRNA targeting MINCR (knockdown) and siRNA control. (**B**,**C**) Relative expression of miR-28-5p and RAP1B mRNA by RT-qPCR after MINCR knockdown in C33-A/AA-c cells. As endogenous controls, RNU44 and GAPDH were used for miR-28-5p and RAP1B, respectively. (**D**,**E**) Transwell cell migration assay. C33-A/AA-c cells were previously transfected with siRNA targeting MINCR and incubated in the transwell chamber for 48 h. The number of migrating cells was determined with ImageJ software. Student’s *t*-test, **** *p* < 0.0001; Mann–Whitney–Wilcoxon test, #### *p* < 0.0001.

To demonstrate that MINCR regulates RAP1B expression through miR-28-5p, MINCR knockdown through siRNAs was performed in C33-A/AA-c cells (Figure 4A). The expression of miR-28-5p and RAP1B mRNA was determined by RT-qPCR after silencing MINCR. As expected, after MINCR knockdown, the expression of miR-28-5p increased 90% compared to the siRNA negative control, while the expression of RAP1B mRNA decreased 60% (Figure 4B,C). These results strongly suggest that MINCR acts as a ceRNA to inhibit the activity of miR-28-5p on its target RAP1B. Finally, to investigate whether MINCR regulates cell migration through miR-28-5p on C33-A/AA-c cells, transwell migration assays were performed. The results show that MINCR knockdown suppressed the cell migration of C33-A/AA-c cells by almost 50% (Figure 4D,E). These results indicate that the MINCR/miR-28-5p/RAP1B axis might have an important role in cervical carcinogenesis in the presence of the HPV16 variant AA-c, mainly by promoting cell migration.

## 4. Discussion

lncRNAs are important molecules during cancer development; however, the molecular mechanisms of many of them remain unknown. The lncRNA MINCR is overexpressed in some cancers such as colon cancer, hepatocellular carcinoma, and non-small cell lung cancer, in which it has been suggested as a potential diagnostic and prognostic biomarker [18,23,24]. However, its role in CC is unknown, as is whether persistent high-risk HPV infection might affect its expression. Therefore, we aimed to decipher the functional role of MINCR in CC using an in vitro model of CC cells with exogenous expression of the E6 oncoprotein of the AA-c variant of HPV16. The results contribute to the knowledge of the molecular mechanisms used by the E6 oncoprotein of HPV16 and its variants to modulate its oncogenic potential.

Previously, our working group reported that the E6 oncoprotein from HPV16 variants differentially regulates gene expression in transfected C33-A cells [9]. We used the transcriptome data from this report to analyze the differential expression of lncRNAs and found that C33-A/AA-c cells overexpress the lncRNA MINCR compared to the control. There are no previous reports of the expression of MINCR in CC; therefore, this finding might mean a new oncogenic mechanism of the E6 oncoprotein of HPV16. Additionally, it could be related to the oncogenic potential of the AA-c variant of HPV16. It has been reported that E6 modulates the expression of some important genes in cancer through proteins such as MYC. The dimer E6/MYC induces hTERT promoter activation, histone modifications, and RNA Pol II phosphorylation [25,26]. These reports suggest that E6 modulates the expression of MYC target genes, among which is the lncRNA MINCR. Interestingly, MINCR silencing reduces MYC transcriptional activity, suggesting positive autoregulation [27].

The role of MINCR has been studied in some types of cancer, where it has been found to inhibit the activity of some miRNAs through the ceRNA function [24,28,29,30]. For example, in bladder cancer cells, MINCR regulates the expression of EZH2 through miR-26a-5p, while silencing MINCR decreases cell proliferation and invasion and increases apoptosis [31]. It has also been reported that, in NSCLC and colorectal cancer, MINCR overexpression promotes cell proliferation and decreases apoptosis [17,24,32].

To test the hypothesis that MINCR acts as a ceRNA in CC, bioinformatic analysis was performed to identify possible target miRNAs. Twelve candidate miRNAs were identified, among which miR-28-5p has been reported to modulate the proliferation in lymphocytic leukemia, prostate cancer, and colorectal cancer [33,34,35]. On the contrary, it has been reported that in endometrial cancer cells, the lncRNA LOXL1-AS1 sequesters miR-28-5p and induces the overexpression of its target RAP1B. RAP1B is a member of the RAS family, which regulates some pathways involved in proliferation, differentiation, and apoptosis [16].

RAP1B has been reported to have a positive effect on the progression of some types of cancer. For example, in lung adenocarcinoma, overexpression of RAP1B induces activation of the Wnt/β-catenin pathway [21], while in colorectal carcinoma, the silencing of RAP1B has an anti-proliferative effect [36], and in esophageal carcinoma, the overexpression of RAP1B is associated with higher levels of proliferation and invasion [37]. Our data show that overexpression of RAP1B in C33-A/AA-c cells could be related to the oncogenicity of the AA-c variant of HPV16; however, the mechanism of the oncoprotein E6 on RAP1B must be investigated.

With this background, we decided to evaluate the MINCR/miR-28-5p/RAP1B axis in C33-A/AA-c cells. The expression of miR-28-5p and RAP1B was consistent with the MINCR expression levels. A negative correlation between MINCR and miR-28-5p and a positive correlation between MINCR and RAP1B were observed. Interestingly, after MINCR knockdown, an increase in miR-28-5p expression levels and a significant reduction in RAP1B expression were observed. These results strongly suggest that MINCR regulates RAP1B expression through targeting miR-28-5p. Importantly, MINCR knockdown decreases cell migration by almost 50%, which is consistent with reports for other types of cancer.

Some reports have indicated that RAP1B promotes cell invasion and metastasis through the regulation of integrins and cadherins, which suggests that RAP1B is involved in the control of adherens junctions [38]. In thyroid cancer cells, miR-200b/c inhibits the activity of the NF-κB/Twist-1 pathway and suppresses cell migration and invasion by reducing RAP1B levels [39]. In some types of tumors, RAP1B is overexpressed, and its inhibition decreases cell proliferation and migration. In addition, RAP1B increases the transcriptional activity of Twist-1, which is an EMT-inducing transcription factor during tumorigenesis [40,41]. In pancreatic cancer, Twist-1 promotes metastasis by interacting with EZH2 and downregulating the expression of proteins such as E-cadherin and p16 [42]. In CC, the molecular function of RAP1B has not been elucidated; therefore, more experiments are needed to clarify the role of RAP1B in the cell migration of the CC-positive AA-c variant of HPV16.

**Figure 5:**
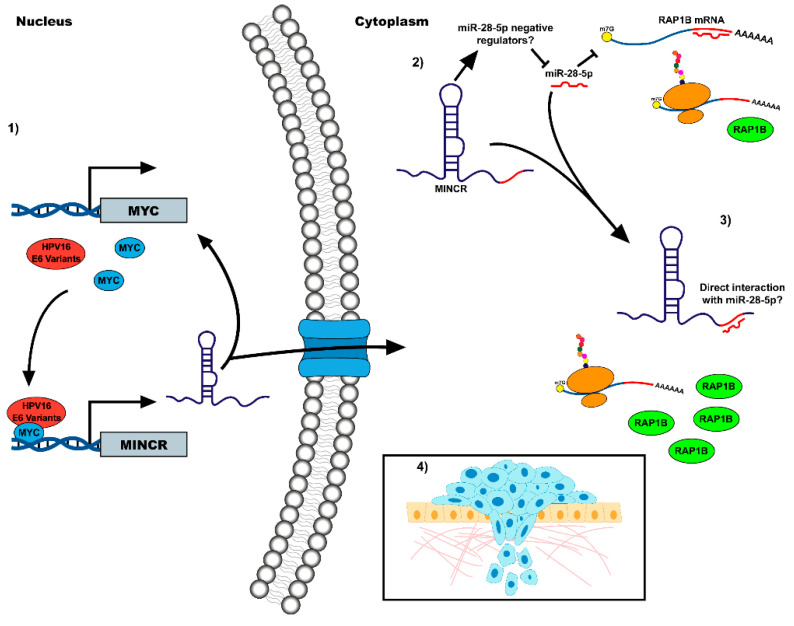
Schematic model of the mechanisms of the HPV16 oncoprotein E6 on the MINCR/miR-28-5p/RAP1B axis. (**1**) Through its interaction with the transcription factor MYC, the oncoprotein E6 activates the expression of MYC target genes, including MINCR. The expression of the HPV16 E6 oncoprotein variants in a cervical cancer model induces overexpression of MINCR, which is involved in the control of the MYC transcriptional network, thus generating a positive feedback between both genes. (**2**) In the cytoplasm, MINCR inhibits miR-28-5p activity on RAP1B mRNA through some possible mechanisms: the direct interaction of MINCR with the miRNA or the activation of negative regulators of miR-28-5p. (**3**) A reduction in the activity of miR-28-5p on its target mRNAs allows an increase in RAP1B mRNA and protein. (**4**) RAP1B overexpression promotes cell migration in cervical cancer cells.

In this work, we reported, for the first time, that the MINCR/miR-28-5p/RAP1B axis positively regulates cell migration in CC-derived cells that express the E6 oncoprotein of the AA-c variant of HPV16. Our results suggest a new oncogenic mechanism of HPV16, through the action of a lncRNA that could then be proposed as a possible therapeutic target in the treatment of CC (Figure 5).

## Supplementary Materials

The following supporting information can be downloaded at: https://www.mdpi.com/article/10.3390/v14050963/s1, Table S1. MINCR target miRNAs; Table S2. miR-28-5p targets.
