# Supplementary material for: The E6 Oncoprotein of HPV16 AA-c Variant Regulates Cell Migration through the MINCR/miR-28-5p/RAP1B Axis
Source: Viruses. 2022 May 5;14(5):963. doi: 10.3390/v14050963 (PMC9143115; doi:10.3390/v14050963)
Supplement: Supplementary file 1 [file viruses-14-00963-s001.zip › viruses-1692406-supplementary.pdf]

Supplementary Table S1. MINCR target miRNAs

miRNAname	miRNAid	merClass	PancancerNum
hsa-miR-374a-3p	MIMAT0004688	7mer-m8	4
hsa-miR-513a-5p	MIMAT0002877	7mer-m8	2
hsa-miR-223-3p	MIMAT0000280	7mer-m8	16
hsa-miR-421	MIMAT0003339	8mer	2
hsa-miR-3167	MIMAT0015042	7mer-m8	1
hsa-miR-876-5p	MIMAT0004924	7mer-m8	2
hsa-miR-28-5p	MIMAT0000085	7mer-m8	4
hsa-miR-708-5p	MIMAT0004926	7mer-m8	4
hsa-miR-3139	MIMAT0015007	7mer-m8	1
hsa-miR-146a-5p	MIMAT0000449	7mer-m8	10
hsa-miR-146b-5p	MIMAT0002809	7mer-m8	8
hsa-miR-7153-5p	MIMAT0028216	7mer-m8	0

Supplementary Table S2. miR-28-5p targets

Gene symbol	Reference Sequence id	Number of pairings	Position
TEX261	NM_144582	19	3UTR
N4BP1	NM_153029	19	3UTR
RAP1B	NM_015646	8	3UTR
